# Burrow emergence rhythms of *Nephrops norvegicus* by UWTV and surveying biases

**DOI:** 10.1038/s41598-021-85240-3

**Published:** 2021-03-11

**Authors:** Jacopo Aguzzi, Nixon Bahamon, Jennifer Doyle, Colm Lordan, Ian D. Tuck, Matteo Chiarini, Michela Martinelli, Joan B. Company

**Affiliations:** 1 Instituto de Ciencias del Mar (ICM-CSIC), 08003 Barcelona, Spain; 2 Stazione Zoologica of Naples (SZN), 80122 Naples, Italy; 3 Marine Institute (MI), Oranmore, Galway H91 R673 Ireland; 4 National Institute of Water and Atmosphere (NIWA), Auckland, 1010 New Zealand; 5 Department of Biological, Geological and Environmental Sciences, University of Bologna, 40126 Bologna, Italy; 6 Institute of Marine Biological Resources and Biotechnologies, National Research Council (CNR IRBIM), 60125 Ancona, Italy

**Keywords:** Ecology, Ecosystem services

## Abstract

Underwater Television (UWTV) surveys provide fishery-independent stock size estimations of the Norway lobster (*Nephrops norvegicus*), based directly on burrow counting using the survey assumption of “one animal = one burrow”. However, stock size may be uncertain depending on true rates of burrow occupation. For the first time, 3055 video transects carried out in several Functional Units (FUs) around Ireland were used to investigate this uncertainty. This paper deals with the discrimination of burrow emergence and door-keeping diel behaviour in *Nephrops norvegicus,* which is one of the most commercially important fisheries in Europe. Comparisons of burrow densities with densities of visible animals engaged in door-keeping (i.e. animals waiting at the tunnel entrance) behaviour and animals in full emergence, were analysed at time windows of expected maximum population emergence. Timing of maximum emergence was determined using wave-form analysis and GAM modelling. The results showed an average level of 1 visible *Nephrops* individual per 10 burrow systems, depending on sampling time and depth. This calls into question the current burrow occupancy assumption which may not hold true in all FUs. This is discussed in relation to limitations of sampling methodologies and new autonomous robotic technological solutions for monitoring.

## Introduction

The Norway lobster, *Nephrops norvegicus* (L.), is one of the most commercially important fisheries in Ireland and also Europe^1^. The 2019 EU Total Allowable Catch (TAC) for *Nephrops*^2^ for the north east Atlantic Functional Units (FU) was close to 44,000 tonnes, and valued at approximately 360 million EUR in 2016^3^. Traditional fishery-dependent sampling methods such as commercial trawling provide indirect biomass estimates of exploited stocks, by means of abundance indices derived from surface density data (i.e., the number of animals per haul-swept area^4–6^).

However, animals construct and inhabit burrow systems used for shelter and for territorial control^7^ and are not available for trawl capture when hiding in the substrate^8,9^. The burrow emergence rhythmicity of populations causes marked fluctuations in catch rates over the 24-h^10^. Peaks in trawl Catch Per Unit Effort (CPUE) shift in timing with increasing fishing depth^11–13^: from full night to dusk- dawn transitions, going from upper to middle-lower shelves, to be finally fully diurnal (i.e. at midday) on upper and middle slopes. This indicates that the species sets its timing of burrow emergence upon a maximum illumination threshold that varies on the depth axis, based on the differential penetration of light as the sun progresses through its diurnal trajectory^10,14^.

The diel rhythm of burrow emergence is more complex than previously thought and it can be subdivided in three different phases^11,15^ : Full emergence, full retraction and an intermediate period in which individuals wait at the burrow entrance (i.e. door-keeping^16^). To date, the proportion of animals not emerging from their burrows on a daily basis is still largely undetermined, although acoustic tagging of individuals of a philetically closely related species has offered some insight^17^. In addition to environmental light, other ecological reasons seem to modulate the predisposition of individuals toward emergence or retraction. For example, crustaceans are at intermediate levels of the marine food webs and their feeding activity (coinciding with burrow emergence in the case of *Nephrops*) is the product of a mortality risk ratio between hunger state and chances to meet visual predators^18,19^.

Alternative fishery-independent assessment methods as Underwater Television (UWTV) surveys using towed camera-sledges, have been developed to estimate stock abundance^20,21^. Those video-based surveys are carried out in several European countries and are coordinated through the International Council for the Exploitation of the Sea Working Group on *Nephrops* Surveys (WGNEPS)^1,22,23^. This more direct (i.e. image-based) method of assessment counts burrow systems, based on their characteristic structural features (i.e. large crater-like entrances^4,24,25^ and their characteristic arrangement individual burrow entrances^20^. Those systems are composed of multiple entrances, shafts and tunnels and can be readily identified by classic features and orientation of the individual burrow entrances, where the apexes of those entrances facing each other in a simple U-shaped system or converging on one central point in a more developed system (i.e. T-shape)^26,27^. This method is independent from the time of the day and season. The burrow system counts can be used as a relative or absolute index for determination of *Nephrops’* stock status and together with catch data can provide a Harvest Rate (HR; catch in numbers/burrow numbers)^28, 29^.

To use UWTV burrow abundance to calculate catch and landings derived from an acceptable Harvest Rate (HR = catch in numbers/UWTV abundance) it is necessary to adjust using agreed correction factor which takes into account the bias associated with UWTV surveys. The key bias contributions for individual *Nephrops* Functional Unit (FU) have been documented and the cumulative correction factor considers edge effect, burrow identification, burrow detection and burrow occupancy^28,29^.

Given the strong territorial behavior of the species, burrow counting seems to be a good proxy for local population abundance, assuming the condition “one burrow system, one animal”^30^, which is the current assumption for *Nephrops* stock assessment^28,29^. The burrow system acts as the center of a strong territorial rhythmic behavior^7,31,32^ and two adult lobsters are rarely found in the same shelter^33^. No spatial segregation occurs between juveniles and adults^34^ and the majority of juveniles build adult-juvenile burrow complexes, which become separated as juveniles grow, and each individual develops its own section^35^. However, burrows systems could also be inhabited by other benthic fish and crustacean species or may remain empty and intact for an unknown period of time after the animals’ death (e.g. due to fishing or natural mortality^30,36^). These factors still pose uncertainties about the true numbers of animals occupying video-counted burrow systems, representing a problem when using UWTV data in stock assessment models^30^.

To improve knowledge of the stock assessment assumption “one burrow system, one animal” a precise temporal description of burrow emergence rhythmicity could be provided by temporally distributed transects within UWTV surveys, similar to that explored with trawl data^11^. The sum of animals engaged in both behaviours could then be compared with counted burrows at phases of maximum population emergence. Unfortunately, temporally scheduled UWTV operations have never been systematically performed and the analysis of rhythmic fluctuations in video-observed animals performing full emergence and door-keeping is not yet available.

Here, we used UWTV survey data reporting densities of full emergence and door-keeping animals and burrow systems from more than three thousand video transects, conducted in the past decade around Ireland, to temporally define both behaviours and their reciprocal relationship over the 24-h. Results on estimated densities of animals engaged in full emergence and door-keeping were then compared with burrow system density estimates, to provide a comparison to the stock assessment assumption “1 burrow system, one animal”.

## Materials and methods

### The study areas and the UWTV surveys methodology

**Figure 1:**
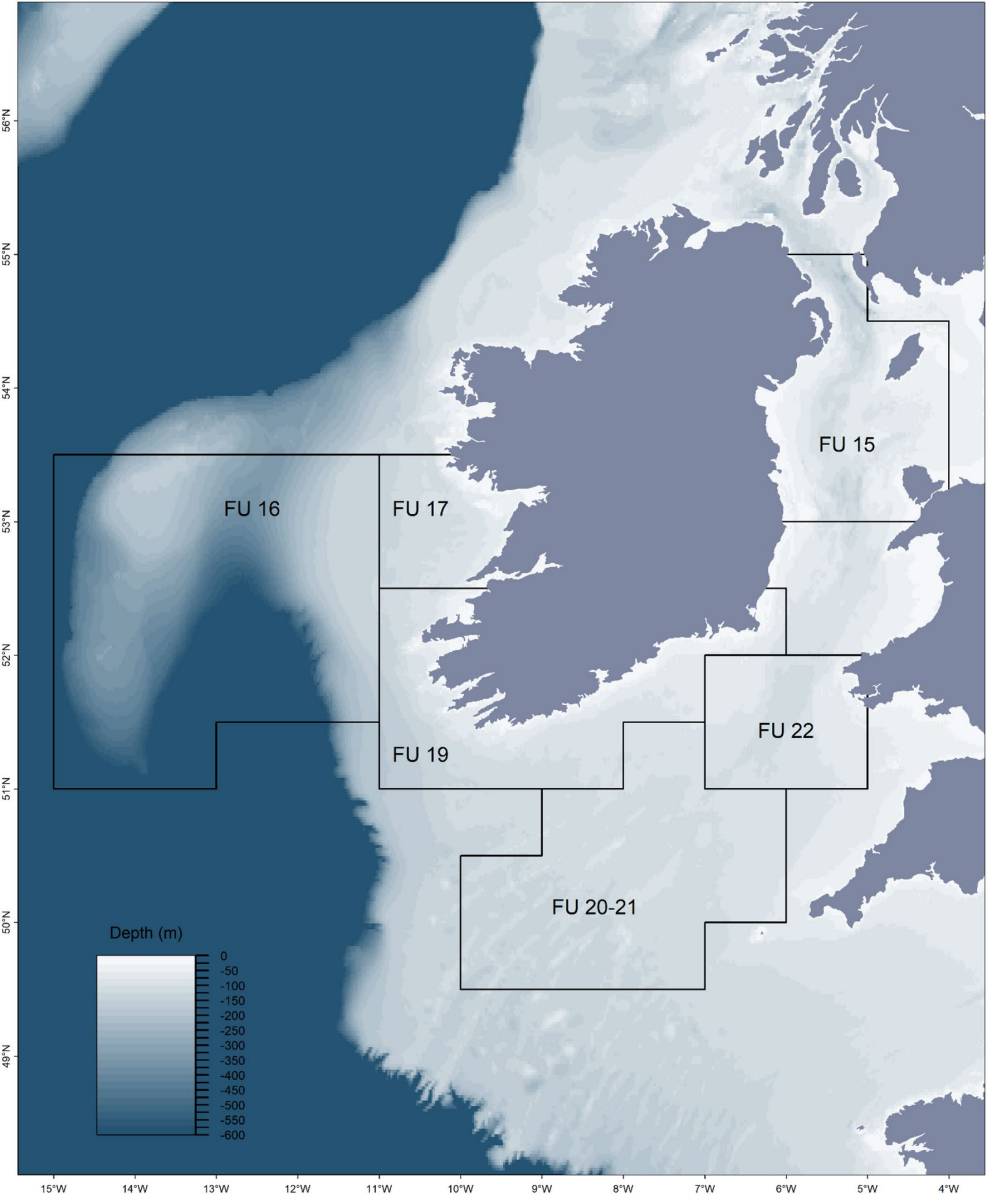
The UWTV survey areas around the Irish coast. Numbers represent the ICES *Nephrops* Functional Units (FUs), as defined by the ICES *Nephrops* Working Group. Depth (m) for the study areas (GEBCO bathymetry data). Map created using: R version 3.61 (2019–07-05) software, https://tinyurl.com/yy6rzlut.

Video footage and derived data were collected from 3055 UWTV transects conducted from 2002 to 2013 in FU areas in the seas around Ireland (Fig. 1). Footage from each transect for all survey areas had a minimum recorded duration of 10 min. The counted minutes of each transect was in line with the prevailing international counting procedure; in years 2002 to 2008 10 min were counted, and in years 2008 to 2013 7 min were counted^28,29^. For FU 16, 10 min were counted for all years, due to the lower densities observed and the relative scale of variation between minutes was higher than typically found in other areas. All considered data were collected in spring–summer surveys (from May to September) (Table 1), in order to avoid variations in the number of video-counted animals and based on reproductive and moulting cycles (see next section).

**Table 1:** List of *Nephrops* functional units (FU), including their code number, area name and depth range.

FU code	Area	Depth range (m)		Sampling grid spacing	Sampling months					Number of UWTV transects
		Min	Max	(km)	May	Jun	Jul	Aug	Sep	
15	West Irish Sea	15	162	5.0				X	X	1501
16	Porcupine Banks	343	570	6.0		X		X	X	115
17	Aran grounds, Galway Bay, Slyne Head	26	162	3.5	X	X	X	X		854
19	South and SW coasts of Ireland	18	116	–		X	X	X	X	101
20–21	Labadie, Jones and Cockburn Banks	95	138	6.0			X	X	X	125
22	The Smalls	74	145	4.5		X	X			635

FU grid spacing is shown for randomised isometric sampling designs. Random stratified sampling is used for FU19. The distribution of UWTV surveys across summer months and number of transects in each FU is also shown.

Sampling design followed either a randomised isometric grid with a station spacing dependent on the individual survey area or a random stratified design^37^ (Table 1). The initial ground perimeter was established by using a combination of integrated logbook-VMS^38^ and habitat data (see methods described in Ligas et al.^39^). The final perimeter has been established using an adaptive approach where stations were located beyond the previously known perimeter of the ground, until the burrow system densities were zero or very close to zero. Once established, the survey area was not changed between years.

At each station, the UWTV sledge was deployed and once stable on the seabed, a 10 min’ tow was recorded onto DVD. The field of view of the camera (Kongsberg OE14-366) at the bottom of the screen with the sledge flat on the seabed (i.e. no sinking), was validated at 75 cm by two parallel spot lasers. Vessel position (by Differential Global Positioning System-DGPS) and sledge position (using an on-board Ultra Short Baseline-USBL, transponder) were recorded every 2 to 5 s. USBL navigational data were used to calculate the video transect distance over the ground, as required for surface density estimates (see next section). The navigational data were quality controlled using an “R” script developed by the Marine Institute^28,29^.

### Data processing

The same footage was viewed and counted by two scientists independently of each other and burrows were identified based on key structural features from an established set of classification keys^20,25,40^. All scientists were trained prior to counting following the ICES recommendations^28,29^, in such a way the counters can be quite consistent in the recognition of a *N. norvegicus* burrow as compared to other species. Final burrow densities were based on an average from the two independent counts after passing quality control processes such as screening for outliers and use of Lin’s concordance CCC to evaluate counter performance^20^. The quality assured burrow density values were then corrected for stock specific survey cumulative bias as described in ICES^29^.

Video-counts of door-keeping animals, defined as those with partial cephalothorax or claws were visible across the burrow mouth entrance, and animals in full emergence which were entirely visible were available^10,15^. As with burrow systems, densities of animals in door-keeping and full emergence were obtained for each transect by dividing respective counts by the video-swept surface.

Each density estimate for burrow systems and animals in both behavioural categories was associated to a time stamp, represented by the time at mid transect length. All density data were grouped per depth ranges within the upper and lower shelf, based on the previous knowledge from trawl catch patterns^11,41,42^ nominally as: 15–50, 51–100, 101–160 and 340–570 m. No data were available for 161–339 m depth. Data for the bathymetric range 340–570 m were only available in FU 16 for the years 2012 and 2013, and this inclusion was necessary to characterize behavioural rhythms in the deepest range for comparison with shallow shelf observations (as previously done with trawling^11^).

The rational for those depth groupings was that *Nephrops* burrow emergence is an adaptive life trait under strong selection which can be temporally described as different on upper and lower shelves as well as slopes^11^. Moreover, the burrow emergence rhythm manifests itself similarly in all its geographic range, with coincident nocturnal, crepuscular or diurnal timings according to the depth light driven peaks not blurred by the tidal status^10,43^. This behaviour is constant through years, subjected only to a seasonal reproduction and growth pattern (e.g. berried females do not emerge^15,44,45^), the effects of which were eliminated here by selecting only summer data (see Table 1).

### Statistical analysis

Firstly, we ran a waveform analysis to describe averaged full emergence and door-keeping behavioural rhythms over the 24-h within the established depth ranges (see above). Waveforms plots describe the phase of a rhythm as an averaged peak into a time series of density data for both behavioural categories. Waveform computing procedure was as follows^46^. A standard day was divided into 1-h intervals and all density estimates for animals in full emergence and door-keeping were pooled together from all the surveys within the same depth range and then averaged at corresponding 1-h timing.

The resulting set of averaged density estimates were then represented over the 24-h with their standard deviations, plus the Midline Estimated Statistic of a Rhythm (MESOR^47^). MESOR is a re-average of all waveform values to be represented onto waveform plots as a horizontal threshold line. All waveform values above the threshold delimit the duration of the peak (i.e. activity peak duration^46^). Waveforms for full emergence and door-keeping density estimates were plotted together to highlight their temporal relationship.

Then, we fitted Generalised Additive Models (GAM) onto full emergence and door-keeping data for spring–summer at the established depth ranges (see above), to achieve statistic formalization of observed emergence patterns beyond data variability (Appendix 1). The package ‘mgcv’^48^ in R^49^ was used with the restricted maximum log-likelihood approach. The effects of the inter-annual variability and the variability among FUs were assessed in the models. The Hour of the Day (HD), from zero to 23 h, was the covariate used to characterize behavioural rhythms. The day-length and the average location of the transects (latitude and longitude) were adopted as spatiotemporal covariates in the full model, following the form:

1 $$E\left(NEP\right)={g}^{-1}\left({\beta }_{0}+year+ FU+ s\left(HD, bs={\text{cc}}, k=24\right)+s\left(Daylength\right)+ te\left(Lat, Lon\right)\right)$$

where *E(NEP)* is the Expected value of *Nephrops* full emergence or door-keeping, conditionally distributed according to the Gamma distribution family. *g* is the log link function, ${\beta }_{0}$ is the intercept. *s* is the smoothing function with the term *bs* = "cc" specifying the 24 h’ knot based (k = 24) cyclic cubic regression spline. The day-length was estimated as the difference between the sunrise and sunset times. *te* is the tensor smooth function for the interaction among transect locations (i.e. latitude and longitude) accounting for spatial dependence on diel activity rhythms affecting *NEP*. Alternatively to the *te(Lat, Lon)* effect, the potential effect of the station locations per FU, *te(Lat, Lon, by* = *FU),* and the interaction between the station locations with the year survey *ti(Lat, Lon, year, d* = *c(2,1)*, were also tested in the models. The *ti* tensor product spline tested the significance of the space-year interaction effect. The 2-dimensional space and the 1-dimensional year factor were specified with the argument *d* = c(2,1).

The models showing significant HD term (i.e. behavioural rhythm), and other significant covariates substantially improving the model variance were selected as the final models (see Appendix 1). The different models were fitted and compared using the percentage of explained deviance and the Akaike Information Criterion (AIC) to select the best one^50^. The range of AIC values of models within depth ranges was generally narrow and not assumed to be critical for the final model choice. The selected final model followed the form (see Appendix 1):

2 $$E\left(NEP\right)={g}^{-1}\left({\beta }_{0}+s\left(HD, bs={\text{cc}}, k=24\right)+f(Cov)\right)$$

where *f*(*Cov*) represented the term *te(Lat, Lon)* for the emergence and door keeping behaviours in the upper depth range (15–50 m). In the case of the emergence behaviour at the depth ranges between 51 and 160 m and door keeping at 101-160 m, *f*(*Cov*) represented the term *s(Daylength)*. Because of the indistinguishable effect of the terms *te(Lat, Lon)* or *s(Daylength)*, on the *NEP* behavioural pattern (see Appendix 1), here we show results and focus on the behaviour produced by the HD term of *NEP*.

We averaged over periods of 1-h, and this probably resulted in models with relatively less variable behaviour during the day, even if the general distribution of *Nephrops* in full emergence and door-keeping did not show appreciable changes. The depth range models allowed identifying peaks timing and duration of full emergence and door-keeping behaviours from the fitted values above the mean.

Finally, in order to identify the temporally optimum moment to count the highest number of individuals (i.e. those in full emergence plus those in door-keeping) in relation to burrow counting, hence obtaining a best estimate of burrow occupancy, a temporally integrated chart of all waveform and GAM results phases^48,49^ was created. Peaks were represented together as continuous horizontal lines and plotted in order to achieve an overall perspective of their temporal relationship^51,52^.

## Results

**Figure 2:**
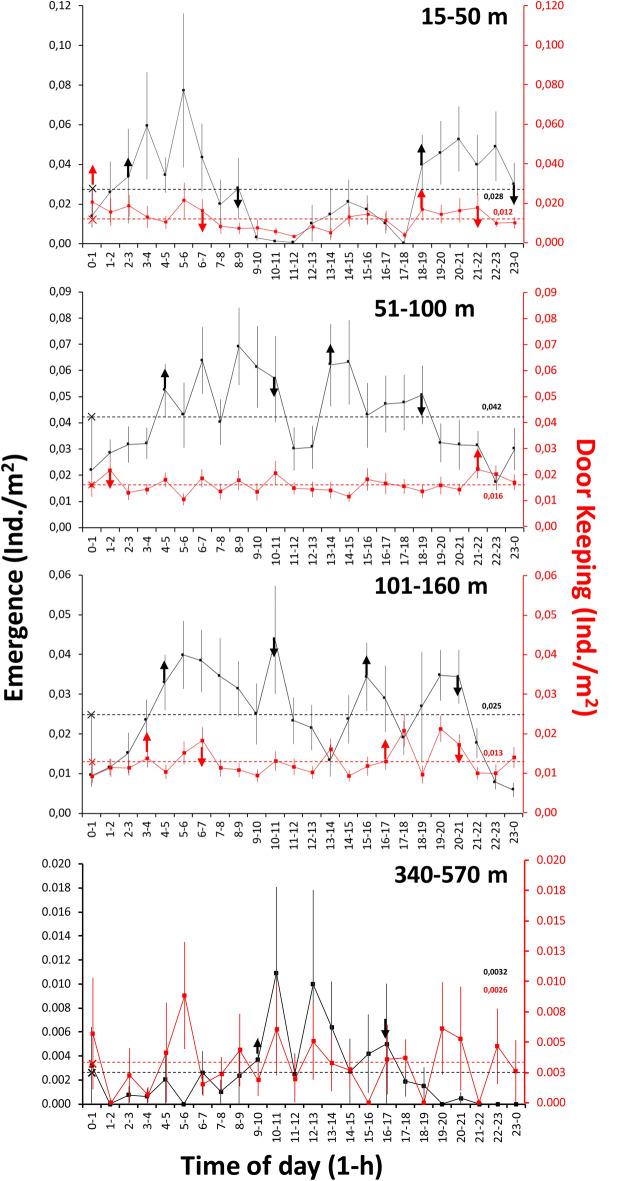
Waveform analysis results depicting the change in burrow emergence behaviour upon depth in terms of full emergence (black) and door-keeping (red). MESORs are the threshold horizontal dashed lines (respective values are also reported with corresponding colour), which identify peak temporal limits (i.e. values above it; coloured vertical arrows). The peak duration is an indication of global averaged activity for that behavioural component in the population. Separate peaks were identified if 2 or more consecutive points were below the MESOR.

Waveform analysis (Fig. 2) indicated that *Nephrops* full emergence varied from nocturnal toward midday hours with increasing depth of sampling. This pattern is particularly evident, when comparing the two extremes of the sampling depth range: upper shelf (15–50 m depth) with two peaks (hour intervals: 2 to 9 and 18 to 0) *versus* middle slope (340–570 m depth) with single peak (hour interval: 9 to 17). At intermediate sampling depths of the lower shelf (51–100 m depth) and shelf-break (101–160 m depth), a less clear crepuscular (dusk and dawn oriented) pattern was reported, with less distinct peaks merging toward daytime. In contrast, door-keeping behaviour had some defined pattern with crepuscular peaks coinciding with full emergence only on the upper shelf (15–50 m depth) and the shelf-break (101–160 m depth). No defined rhythms were discernible at the other depth zones.

**Figure 3:**
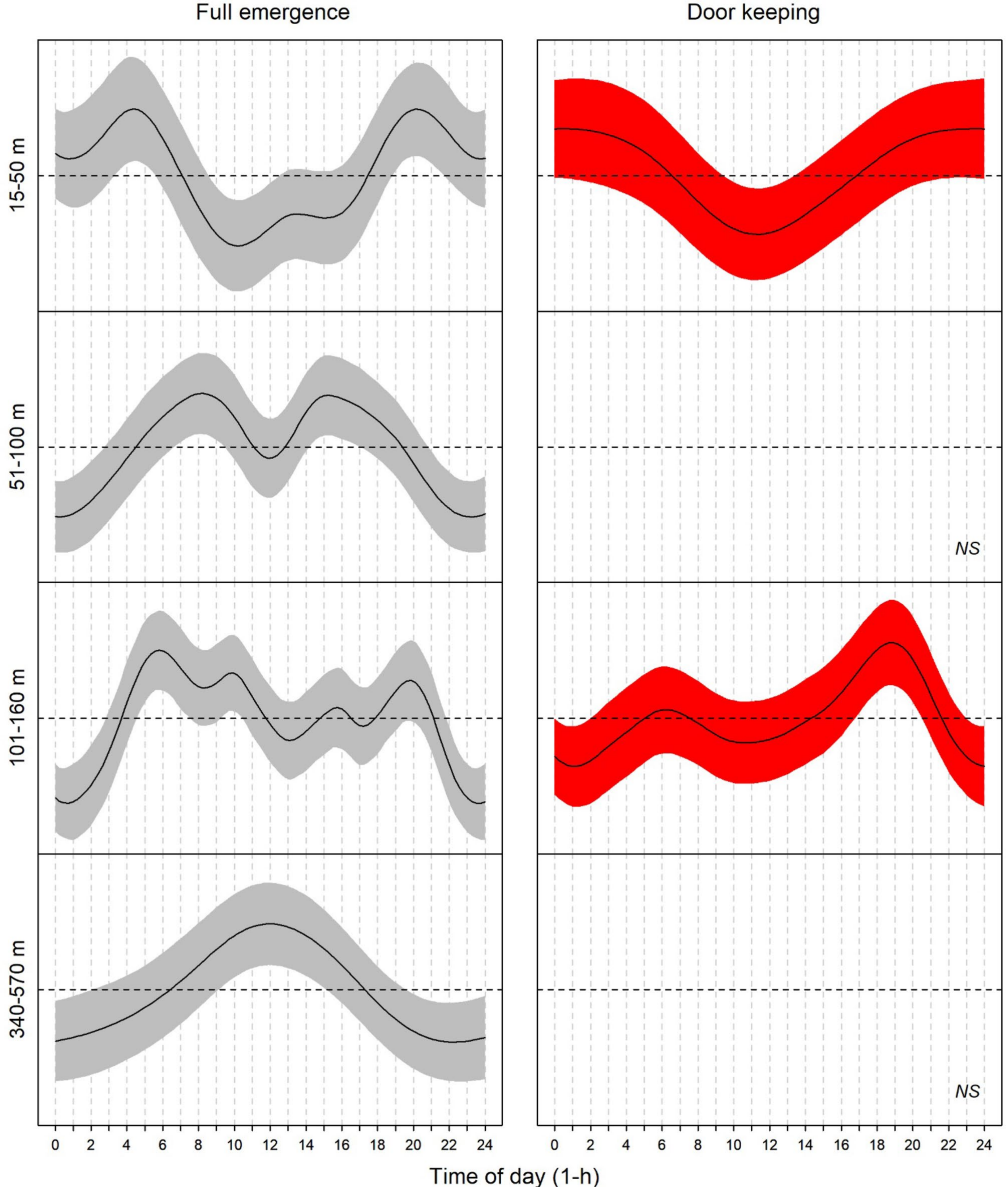
Significant (*p* < 0.05) GAM modeled temporal patterns for full emergence (grey) and door-keeping (red) behaviors by depth ranges over the 24-h cycle. Shadowed areas represent 95% confidence intervals of modelled patterns. Horizontal black dashed lines are the zero-mean values, taken as a reference to estimate representative time ranges of full emergence and door-keeping activity peaks (i.e. values above the mean). Door-keeping model fits at 51–100 and 340–570 m depth ranges were not significant (*p* > 0.05).

The statistical model results by GAM (Fig. 3), revealed an overall pattern of full emergence and door-keeping behaviour similar to that found from the waveform analysis (Fig. 2). In the upper continental shelf (15–50 m depth), the model shows a nocturnal bimodal (i.e. two peaks) emergence pattern (hour intervals: 2 to 7 and 17 to 23). On deeper shelf areas (from 51 to 160 m depth), the emergence pattern becomes diurnal with a plateau shape (i.e. no major crepuscular peaks). Finally, in the upper slope (340–570 m depth) the emergence shows a single peak (hour interval: 7 to 17). Consistent with the waveform analysis (see Fig. 2), door-keeping, showed a less clear temporal pattern (Fig. 3). As with emergence, door-keeping was nocturnal with weak crepuscular increases at 15–50 m depth range (hour intervals: 0–7 and 15–0). The temporal pattern was lost between 51 and 100 m to be regained with a crepuscular aspect on the shelf break (hour intervals: 4–7 and 15–21), becoming again completely arrhythmic on the upper slope.

**Figure 4:**
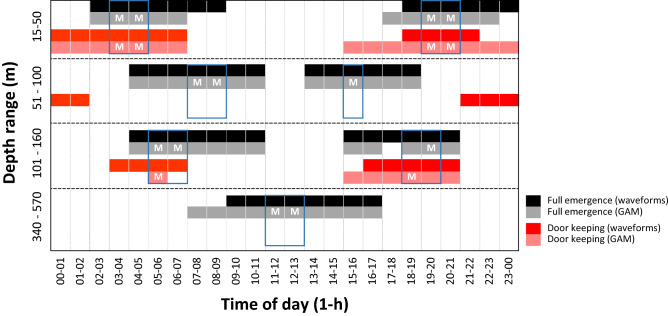
Integrated chart showing temporal relationships among peaks timings, as derived from plots of waveform and GAM analyses (i.e. phases as values above horizontal threshold lines; see Figs. 2 and 3). According to GAMs, the best timings of UWTV surveying where highest (maximum) number of animals can be observed, are indicated with the letter M and grouped by blue rectangles.

The temporal comparison between the waveform analysis (see Fig. 2) and GAM model outputs (see Fig. 3) in peak timings and duration per depth stratum is presented in Fig. 4. In the upper shelf (i.e. 15–50 depth), GAM modelling indicated a slightly shorter timing of nocturnal emergence. At intermediate and lower shelf (from 51 to 160 m depth), the waveform and GAM analysis shows a slight drop of emergence at noon. On the slope (340–570 m depth), the midday timing for emergence indicated for waveform analysis (hour interval: 9–17) was modelled by GAM as taking place for a longer duration (hour interval: 7–17).

The same comparison for door-keeping behaviour (see Fig. 4) showed a nocturnal rhythmicity at depths 15–50 m with both waveform analysis and GAM, with a duration slightly larger for the latter. Although no significant temporal pattern was detected by the GAM modelling from 51 to 100 m depth and from 340 to 570 m depth, on the shelf-break some weak crepuscular temporization was detected by the two analysis approaches.

Independently of the survey time, the maximum densities of emergence and door-keeping were detected in the 51–100 m depth layer (means of 0.058 and 0.020 Ind./m^2^, respectively, in the FU 15, West Irish Sea), coinciding with the maximum number of burrows per area (0.908 burrows/m^2^) (Table 2). This corresponds to a visible occupancy of 0.086 individuals per burrow.

**Table 2:** Time-independent density of animals (Ind./m^2^) at emergence and door-keeping per depth range by *Nephrops* functional units (FUs).

FU code	Depth range (m)	N of UWTV transects	Emergence (Ind./m^2^)		Door keeping (Ind./m^2^)		Burrow density (bur/m^2^)	
			Mean	SD	Mean	SD	Mean	SD
15	15–50	349	0.022	0.054	0.008	0.016	0.622	0.606
	51–100	754	0.058	0.087	0.020	0.025	0.908	0.517
	101–160	398	0.042	0.076	0.017	0.021	0.895	0.458
16	340–570	115	0.003	0.006	0.003	0.006	0.124	0.061
17	15–50	71	0.016	0.031	0.015	0.019	0.849	0.394
	51–100	261	0.015	0.035	0.009	0.017	0.393	0.374
	101–160	522	0.019	0.038	0.015	0.019	0.678	0.354
19	15–50	5	0.000	0.000	0.006	0.010	0.396	0.321
	51–100	55	0.005	0.014	0.003	0.007	0.292	0.276
	101–160	41	0.005	0.012	0.004	0.007	0.379	0.258
20–21	51–100	4	0.010	0.015	0.002	0.004	0.187	0.374
	101–160	121	0.003	0.016	0.003	0.007	0.387	0.287
22	51–100	267	0.009	0.023	0.005	0.010	0.261	0.236
	101–160	368	0.019	0.037	0.008	0.013	0.571	0.331

The timing of the maximum number of animals in full emergence varied between depth strata (Fig. 4, Table 3). The mean densities of animals ranged from 0.024 and 0.061 Ind./m^2^ over the continental shelf (from 15 to 160 m depth), and one order of magnitude lower on the slope (340–570 m depth) with densities between 0.0064 and 0.009 Ind./m^2^.

**Table 3:** Mean density (Ind./m^2^) of *N. norvegicus* and burrow occupancy at periods with the predicted highest (maximum) number of animals displaying full emergence and door-keeping behaviour, as identified in Fig. 4.

Depth range (m)	Period (h)	Emergence (Ind./m^2^)	Door keeping (Ind./m^2^)	Emergence + door keeping (Ind./m^2^)	Fraction of emergence %	Fraction of door keeping %	Burrows (bur/m^2^)	Burrow occupancy (mean Ind/bur ± SD)
15–50	03–04	0.024	0.011	0.034	69.2	30.8	0.579	0.08 ± 0.02
	04–05	0.057	0.013	0.070	81.6	18.4	0.734	
	19–20	0.037	0.010	0.046	79.0	21.0	0.655	
	20–21	0.043	0.012	0.055	78.1	21.9	0.645	
51–100	07–08	0.045	0.015	0.060	74.9	25.1	0.600	0.11 ± 0.01
	08–09	0.061	0.015	0.075	80.2	19.8	0.736	
	15–16	0.054	0.015	0.069	77.7	22.3	0.582	
101–160	05–06	0.034	0.010	0.044	77.5	22.5	0.517	0.08 ± 0.01)
	06–07	0.041	0.017	0.058	71.1	28.9	0.654	
	18–19	0.027	0.015	0.042	63.1	36.9	0.652	
	19–20	0.026	0.018	0.045	59.0	41.0	0.722	
340–570	11–12	0.006	0.007	0.012	45.7	54.3	0.162	0.09 ± 0.02
	12–13	0.009	0.004	0.013	68.3	31.7	0.130	

Focussing on mean density values for all visible animals (combined totals of both emergence and door-keeping behaviours) at peak timing as a proxy of total population densities (Fig. 4), the following observation can be made: the animal density increases from 0,034 to 0,075 Ind./m^2^ over the continental shelf, and from 0,012 to 0,013 Ind./m^2^ over the slope. The fraction of door-keeping animals (i.e. from the total emergence and door-keeping animals) is slightly lower on the continental shelf than on the slope (18–41% and 32–54%, respectively) (Table 3). The number of total animals visible per burrow system ranged from 0,059 to 0,119 Ind./m^2^ across the shelf and the slope.

## Discussion

The present work describes for the first time the diel behavioural rhythms of *Nephrops* in terms of burrow emergence and door-keeping, based on observations in more than three thousand UWTV transects. Populations emergence patterns varied their timing from the shelf to the slope with a timing shift, which is consistent with previous observations based on trawl catch temporal rates (i.e. in capture peaks from nocturnal to crepuscular and then to fully diurnal hours as the depth increases^11^). In contrast, the description of the temporal variation in door-keeping behaviour is an entirely new finding for *Nephrops*, since individuals at the entrance of their tunnel systems are unlikely to be catchable in trawling operations^53^. Here, we provide evidence of arrhythmic fluctuations in counts of animals expressing this behaviour, with relevant counts sparse over the whole 24-h cycle. This points out that the arrhythmia of observations of door-keeping animals could be due to: a behaviour which is in fact arrhythmic in some individual, or that animals retract into the burrows because they perceive approaching sleds. An unknown part of the population may therefore avoid haul capture by a quick withdrawal of individuals into their burrows when trawls approach^54^. Consequently, the number of counted “door-keeping animals” could be dependent upon sledge towing speed (i.e. animals reacting to the approach of the sledge and retreating^25^), as well as the presence of the sledge light system and overall noise. In any case, for trawl gear to efficiently catch Norway lobsters, they have to be fully outside of the burrows.

### Burrow emergence rhythms and the UWTV-based stock assessment assumptions

Estimated densities of visible animals engaged in both emergence and door-keeping behaviours were compared with burrow system counts and derived density estimates, to provide evidence putative biases to the standard stock assessment assumption that “1 burrow system is occupied and maintained by one animal”^20^. Present results suggest a visible individuals’ ratio of around 1 visible *Nephrops* to 10 counted burrows. This result is a general estimate considering all the FUs, with results closer to the 1:1 assumption in some areas (i.e. FU 15). Taken together, our results indicate that there may well be variations in the burrow occupancy across different FUs. In fact, this difference appears to actually be related to the latitude/longitude position rather than the FU as the GAM showed FU to be irrelevant, while sample location (or day length) was influential in the model (Appendix 1). Video-derived animal densities during the consecutive hours were variable and the minimum estimates of stock densities should be derived focusing on the hours at which maximum densities are visible at the surface. Temporal windows at which we reported maximum emergence (plus door-keeping) densities of *Nephrops* can be considered good time periods to compare animals and burrow systems numbers together.

This burrow occupancy assumption has been identified a major uncertainty in UWTV bases assessment approach, particularly when using the UWTV index as an absolute measure of stock abundance^20^. Field observations indicate a more complex behavioural situation where single individuals can inhabit a single or complex burrow system with a variable number of entrances depending on local population densities^30^. At the same time, laboratory studies on aggressive hierarchy show that dominant individuals attempt to evict the subordinates to conquer their burrows nearby^9^. Even in periods of peak emergence it is possible that not all individuals are visible at the surface. Aguzzi et al.^18^ suggested that the predisposition of animals toward burrow emergence depends on the hunger state (and the presence of carrion and prey) and the absence of potential predators or sympatric competitors. Furthermore, laboratory tests on large numbers of individuals indicate that shelf animals may exhibit a differently phased dusk or dawn emergence, possibly to reduce interspecific competing pressure^13^. This matches present results where the temporal patterns in emergence observed in *Nephrops* UWTV transects generally follow those described by trawl catches on the shelf. The environmental factors such as the lunar cycle, tides and bottom currents, whose strength could vary according to the different local topography in different FUs, could also impact on burrow emergence^16^. Depending upon the future spatiotemporal availability of environmental data (to date missing) new variables could also be modelled, to improve the model correction approach.

It is possible that the number of burrow systems are over estimated, for a variety of reasons. Despite the training systems in place to ensure consistency in *Nephrops* burrow identification, the accuracy of burrow identification may vary across FUs. In some areas sympatric fish and other decapod species occupy or even construct burrows with morphology similar to those of *Nephrops*^30^. It is also possible, in environmentally stable lightly trawled grounds, that unoccupied burrows may persist, and appear to be active (clearly inactive systems are not counted). However, most of the grounds in our study are heavily trawled with swept area ratios > 5^28,29^. The number of burrow entrances per counted burrow system may also be variable in different habitat types. It is unlikely that all these factors can fully account for the discrepancy in the animals to burrow count ratios observed here between areas.

Considering our results and the previously accounted sources of uncertainty for the UWTV-based stock assessment equation, our estimation of the general value of “1 Ind./10 burrow correction factor” does call into question the use of UWTV surveys as an absolute index on *Nephrops* abundance. A visible *Nephrops* index does provide a minimum population estimate of those emerging on a diel basis, but may not account for those concealed. The Irish Sea (FU 15) is a very dynamic system with strong bottom currents and highly populated sea bed^28,29^ so the 1:1 assumption could likely hold in that area. At the same time, this assumption could be quite different in FU 20–21, which is less fished and has lower densities of burrow systems. The HR for FU 15 has for long periods been around 20% and that observation clearly invalidates any possibility that our ratio of 0.1 Ind./burrow can be close to the true value (since the local fishery would be catching twice the number of animals/burrows annually). Still that doesn’t mean that the ratio 1:1 is true for all other FUs.

Trawling is a traditional sampling approach for the scientific monitoring of demersal resources but it does not provide data on the behaviour of the target species nor how such a behaviour can influence catchability^51^. UWTV surveying has distinct advantages over trawling, being more ecologically sensitive, causing minimal physical damage to seabed habitats and allowing better behavioural characterization. However, considerable work remains to be done in order test the key assumptions used in the assessments based on UWTV survey programmes.

### The methodological constrains of our study based on input data typology

We chose to group the data for depth ranges and across years rather than keeping at a higher level of granularity, since *Nephrops* behavior is usually highly variable^55^. In fact, the fitted GAMs suggested that there are no significant effects of the survey year (i.e. inter-annual variability) on the emergence or door keeping patterns. The models also suggested that the variability across FUs is not relevant (i.e. significant) in explaining the behavioral patterns.

Additional data sub-grouping based on light data (not available) could have been performed. However, the estimation of any environmental illumination index based on transect timing, geographic position and depth would result in a mere modelling exercise. Factors such as cloud cover, water column primary productivity and turbidity have a significant impact on light scattering and absorbance (i.e. extinction) coefficients^56,57^, and are unavailable at the high spatiotemporal frequency of UWTV transects for all the FU areas considered.

Moreover, *Nephrops* rhythmicity is part of burrowing behavioral life-styles under natural selection in crustacean decapods, which was shown to be expressed independently from contingent variations in background light intensity^58^. The species possesses a biological clock that would ensure a temporally averaged burrow emergence pattern. The biological clock activates the locomotor activity (at the base of burrow emergence), every 12-h or 24-h, depending from the shelf or slope depth stratum considered^13,59^. This rhythmicity is self-sustained since it keeps its period and phase based on an environmental memory of previously experienced environmental light conditions when animals are transferred to laboratory constant conditions (i.e. entrainment upon intensity and photophase duration)^47,60^.

Another source of data variation may be the underlying dynamics of the populations due to recruitment variations, fishing and natural mortality^39^. In the case of the Mediterranean *Nephrops* stocks, fishery overexploitation is not reducing the number of captured animals but the biomass (i.e. animals are getting smaller)^61^. To date, there is no evidence of a similar finding for the Irish Sea. The local stocks are not experiencing declines due to excessive fishing mortality (e.g. FU 15, has continuously yielded ~ 10,000 tonnes of catch for nearly 60 years)^62^. It is feasible that a behavioral mechanism modulating emergence is preserving the populations from the fishery exploitation (see all considerations above).

### Toward a more technologically sustained fishery-independent stock assessment

Towing the UWTV sledge could bias counts of emerged individuals causing them to flee outside the field of view and cause door-keepers to retract inside their burrows^53^. To improve stock assessments, more intensive data collection efforts are needed to collect data for improved models. Data collection may include optoacoustic by multi-beam cameras that should be used in combination with High-Density (HD) imaging from Autonomous Underwater Vehicles (AUVs)^63^ and Internet Operated Vehicles (IOVs) such as crawlers^64^. A complete photomosaic of the targeted parcel describing burrow systems and their reciprocal positioning, should be undertaken as a first step. Only after that, hourly scheduled AUV and IOV’ acoustic sweeps should be continuously performed during consecutive day-night cycles, replicated in different seasons, to picture emerging and door-keeping *Nephrops* and associated predator and prey species under silent and non-light conditions.

In addition, different stand-alone or cabled observatories, holding several seabed and water column sensors for environmental monitoring (e.g. the OBSEA or SmartBay; respectively, https://www.obsea.es and https://www.smartbay.ie/), could be used to picture burrow emergence modulation (for an insight on monitoring network geometry and characteristics see^65,66^). The different observational points could be synchronously used to account for the control of oceanographic and ecological drivers on the burrowing behavior of the species^67^. With such a multidisciplinary demographic, behavioral and environmental approach one may finally derive more accurate stock assessment models, predicting the density of animals that could be sampled with different fishery-dependent and independent tools^67^.

## Conclusions

Our results highlight that *Nephrops* is highly cryptic and has fascinating behavioural patterns that affect its availability to visual as well as capture-based surveys. The temporal treatment of UWTV video data within the chosen depth ranges showed the behavioural pattern of burrow emergence is predominantly dusk and dawn-oriented above 50 m, bimodal and tending to be diurnal between 50 and 100 m, temporally diffused between 101 and 160 m, and finally fully diurnal between 340 and 570 m, partially matching depth-dependent patterns in trawl catch rates. The door-keeping behaviour is only temporally defined above 50 m (being nocturnal) and bimodal with a nocturnal increase between 100 and 160 m. During the hours of maximum peak abundance of visible individuals (summing up the video-counted individuals in emergence and door-keeping behaviours), we have observed that on average there is about 1 visible individual per 10 burrows, at most. This represents the average peak, although there were higher peaks within individual transects. In general, considering all areas together, our ratio is well below that assumed in current stock assessments (i.e. “1 burrow system:1 animal”), suggesting that a high proportion of the population remains cryptic even during periods of peak emergence. This bias should be carefully considered since an undefined number of animals may avoid the sledge at its approach. Further technological development toward optoacoustic technologies and additional effort for calibration and modelling to integrate observations of visible individuals may further improve the utility of UWTV surveys for stock assessment. Four lines for technological based calibration should be foreseen in future stocks monitoring actions: burrow identification, as other sympatric fish and decapod species occupy or even construct burrows with morphology similar to those of *Nephrops*; intraspecific aggressive relationships and hierarchy, where dominant individuals may occupy different burrow systems nearby; emergence enhancement and inhibition depending on hunger state, due to predators’ presence and the quantity of emerging conspecifics at the “rush hour timing”; and finally burrow persistence after an animal’s death, depending on the density of burrow-dwelling species, local hydrographic and fishing pressure conditions.

## Supplementary Information

Supplementary Information.
