# Supplementary material for: Burrow emergence rhythms of Nephrops norvegicus by UWTV and surveying biases
Source: Sci Rep. 2021 Mar 11;11:5797. doi: 10.1038/s41598-021-85240-3 (PMC7970996; doi:10.1038/s41598-021-85240-3)
Supplement: Supplementary file 1 — Supplementary Information. [file 41598_2021_85240_MOESM1_ESM.docx]

**Burrow emergence rhythms of *Nephrops norvegicus* by UWTV and surveying biases**

Jacopo Aguzzi^1,2,^*, Nixon Bahamon^1^, Jennifer Doyle^3^, Colm Lordan^3^, Ian D. Tuck^4^, Matteo Chiarini^5,6^, Michela Martinelli^6^, Joan B. Company^1^

^1^Instituto de Ciencias del Mar (ICM-CSIC), Barcelona, 08003, Spain

^2^Stazione Zoologica of Naples (SZN), Naples, 80122, Italy

^3^Marine Institute (MI), Oranmore, Galway, H91 R673, Ireland

^4^National Institute of Water and Atmosphere (NIWA), Auckland, 1010, New Zealand

^5^Department of Biological, Geological and Agricultural Sciences, University of Bologna, Bologna, 40126, Italy

^6^National Research Council of Italy (CNR), Institute of Biological Resources and Marine Biotechnologies (IRBIM), Ancona, 60125, Italy

*correspondence and requests for materials should be addressed to: jaguzzi@icm.csic.es

**Appendix 1**

Stepwise selection of the best-fitting GAMs Models (Mod). A number of GAMs were fitted to investigate for the effect of the year survey, Functional Units (FUs), day-length and transect coordinates on the *Nephrops* (NEP) behaviour. The models were adjusted separately for each stablished depth range, from 15 to 570 m depth for surveys from data between 2002 and 2013 (**Table A1**). Final models were selected (with covariates in bold; **Table 1A** and **Figure 1A**) based on the significance of the HD term and other significant covariates strongly improving the total model variance. The range of AIC values was relatively narrow within depth ranges and it was assumed not to be critical for model choice. Thus, models were disregarded if showing no significant covariates or showing significant covariates such as year and FU, but not strongly improving the model variance (e.g. Mod 1 to Mod 4 for the emergence behaviour with the depth range 51-100 m). Apart from the significant effect of the Hour of the Day (HD), the contribution of transect locations and day-length to explain the chosen models variability is indistinct (e.g. Mod 10 for emergence behaviour at depth ranges between 51-100 and 101-160, **Figure 1A**). This is probably due to the fact that the correlations between day-length and latitude (*r* = 0.45) and longitude (*r* = 0.58) are relatively high. Note that, even though the correlation values are relatively high and may be redundant in the model, in some models they were tested together to assess for potential effects of the fraction of uncorrelated data. The final model choice only includes the transects location or the day-length (apart from HD); this suggests that the variability among years and the variability among FUs are irrelevant for explaining the behavioural pattern of *Nephrops*. Even the nested effect of the transect locations into the FUs and the interaction between the transect locations and the survey were not significant in the selected models.

**Table A1**. GAMs fitted to explain *Nephrops norvegicus* behaviour (emergence and door keeping) based on the survey year, Functional Units (FU), the day-length and station locations in the Atlantic Sea around Ireland. The *s* is the smoothing function for the hour of the day (HD) and day-length. *bs* = "*cc*" indicates the 24-h knot based (*k* = 24) cyclic cubic regression spline ("*cc*"). *te* is the tensor smoother for the spatial covariates (i.e. latitude and longitude). The *by* = FU argument nested the station locations into FUs. The *ti* tensor product spline tested the significance of space and time interaction. The argument *d* = *c*(2,1) indicates the function that the smooth consists of tensor product between space (2-dimensional smooth for longitude and latitude) and time (1-dimensional term, year). Models 4 to 8 were not fitted for the depth range 340-570 m, because of limited data (i.e. two years’ data for a single FU). Selected models (shown in **Figure 1A**) are indicated with covariance values in bold.

***^a^*** The interaction *te*(Lat, Lon): FU 15 was significantly different from the other interactions (*p*<0.05).

***^b^*** The year 2003 was significantly different from the reference year 2012 (*p*<0.05).

***^c^*** The years 2003 and 2005 were significantly different from the reference year 2012 (*p*<0.05).

***^d^*** The years 2003 and 2004 were significantly different from the reference year 2012 (*p*<0.05).

***^e^*** The FU 20-21 was not significantly different from the reference FU 15 (*p*>0.05).

***^f^*** The FU 19 was significantly different from the reference FU 15 (*p* = 0.009).

***^g^*** The interaction *te*(Lat, Lon): FU 15 and FU 17, were significantly different from the other interactions (*p*<0.05).

***^h^*** The years 2005, 2006, 2008 and 2009 were significantly different from the reference year 2012 (*p*<0.05).

***^I^*** The years 2006 and 2009 were significantly different from the reference year 2012 (*p*<0.05).

***^j^*** The FU 22 was significantly different from the reference FU 15 (*p* = 0.001).

**Figure A1**. Effects of explanatory variables on the emergence and door-keeping behaviour at the different depth ranges, as estimated by the selected GAMs. The model (Mod) number and depth ranges are shown, as indicated in the **Table A1**. The plots were generated using the package ‘mgcv’^48^ in R^49^.

| **Emergence** | |
| --- | --- |
|  |  |
|  | Mod 10: $E\left( NEP \right)=g^{-1}\left( \beta_{0}+s\left( HD, bs=\text{cc}, k=24 \right)+te(Lat, Lon) \right)$ |
| 15-50 m | 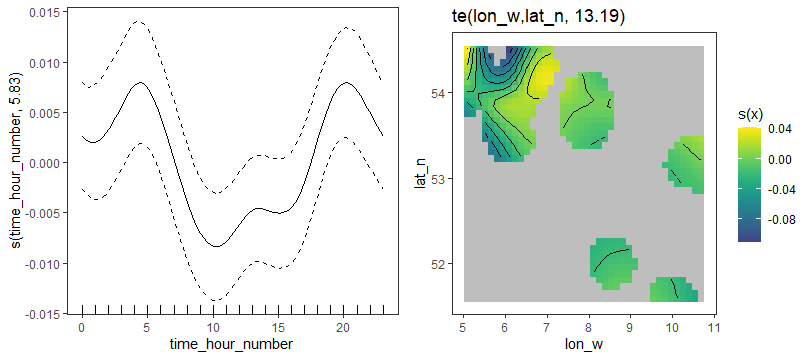 |
|  | Mod 10: $E\left( NEP \right)=g^{-1}\left( \beta_{0}+s\left( HD, bs=\text{cc}, k=24 \right)+te(Lat, Lon) \right)$ |
| 51-100 m | 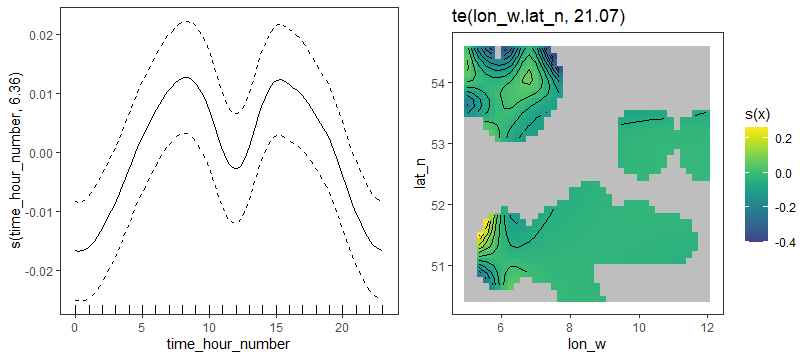 |
|  | Mod 11: $E\left( NEP \right)=g^{-1}\left( \beta_{0}+s\left( HD, bs=\text{cc}, k=24 \right)+s(daylength) \right)$ |
| 51-100 m | 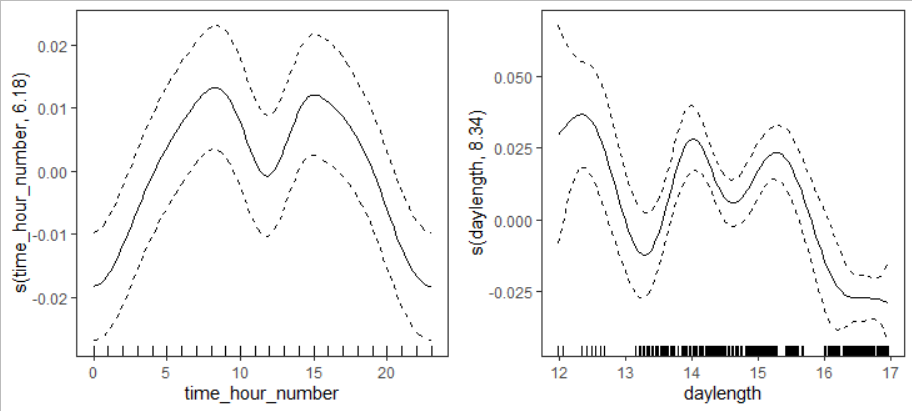 |

**Figure A1**. Cont.

|  | Mod 10: $E\left( NEP \right)=g^{-1}\left( \beta_{0}+s\left( HD, bs=\text{cc}, k=24 \right)+te(Lat, Lon) \right)$ |
| --- | --- |
| 101-160 m | 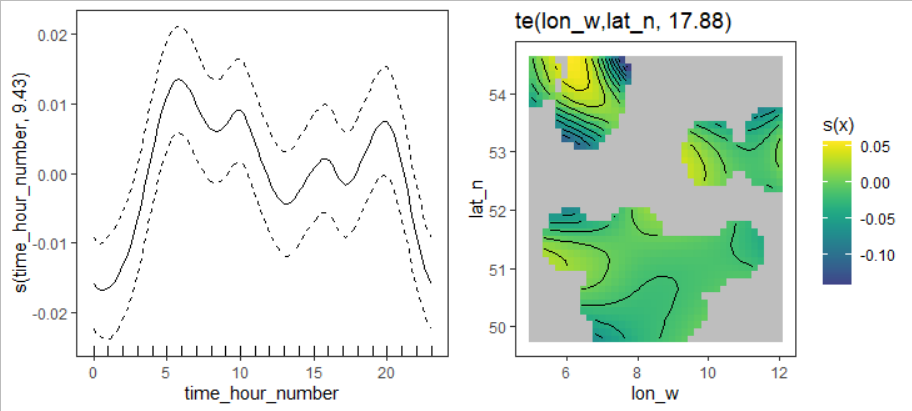 |
|  | Mod 11: $E\left( NEP \right)=g^{-1}\left( \beta_{0}+s\left( HD, bs=\text{cc}, k=24 \right)+s(Daylength) \right)$ |
| 101-160 m | 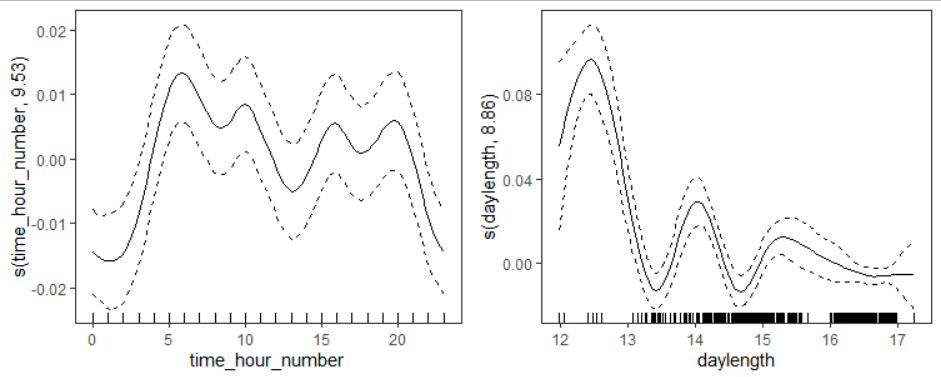 |
|  | Mod 12: $E\left( NEP \right)=g^{-1}\left( \beta_{0}+s\left( HD, bs=\text{cc}, k=24 \right) \right)$ |
| 340-570 m | 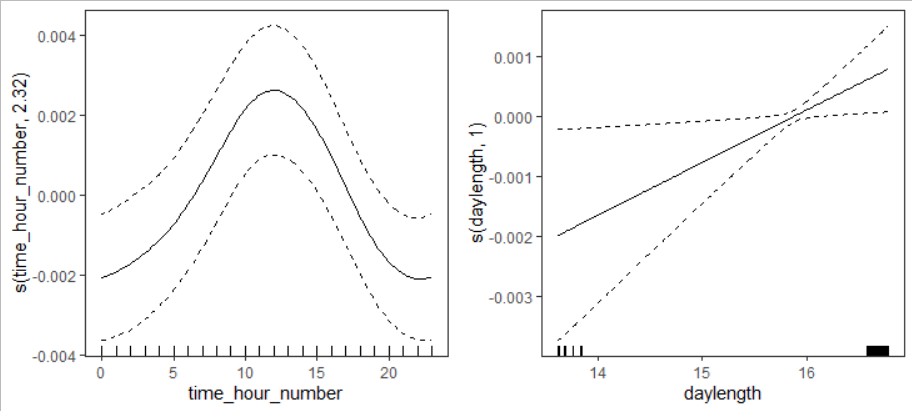 |

**Figure A1**. Cont.

| **Door-keeping** | |
| --- | --- |
|  |  |
|  | Mod 10: $E\left( NEP \right)=g^{-1}\left( \beta_{0}+s\left( HD, bs=\text{cc}, k=24 \right)+te(Lat, Lon) \right)$ |
| 15-50 | 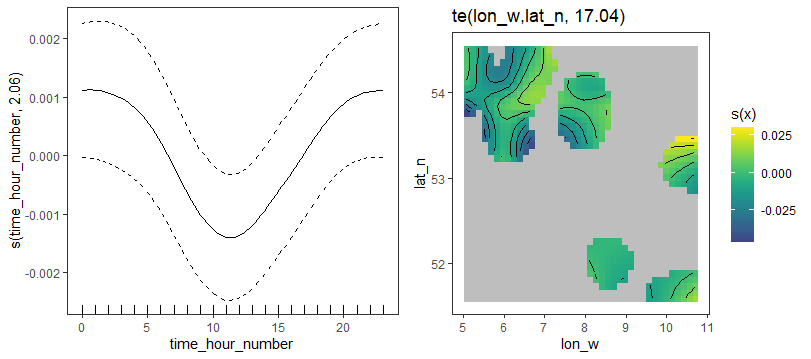 |
|  | Mod 10: $E\left( NEP \right)=g^{-1}\left( \beta_{0}+s\left( HD, bs=\text{cc}, k=24 \right)+te(Lat, Lon) \right)$ |
| 101-160 | 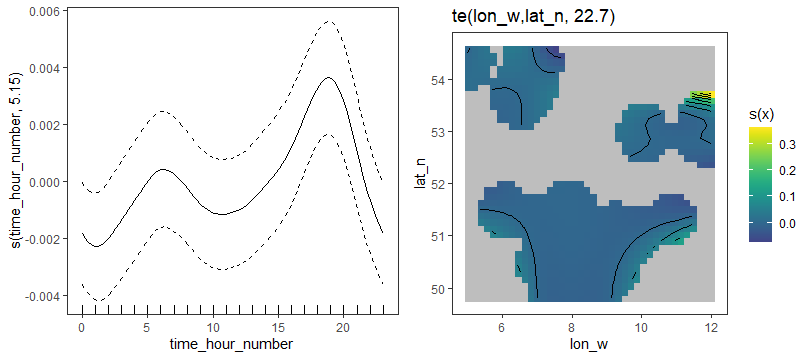 |
|  |  |
